# Iron and Ferritin Modulate MHC Class I Expression and NK Cell Recognition

**DOI:** 10.3389/fimmu.2019.00224

**Published:** 2019-02-26

**Authors:** Rosa Sottile, Giorgia Federico, Cinzia Garofalo, Rossana Tallerico, Maria Concetta Faniello, Barbara Quaresima, Costanza Maria Cristiani, Maddalena Di Sanzo, Gianni Cuda, Valeria Ventura, Arnika Kathleen Wagner, Gianluca Contrò, Nicola Perrotti, Elio Gulletta, Soldano Ferrone, Klas Kärre, Francesco Saverio Costanzo, Francesca Carlomagno, Ennio Carbone

**Affiliations:** ^1^Tumor Immunology and Immunopathology Laboratory, Department of Experimental and Clinical Medicine, University Magna Graecia of Catanzaro, Catanzaro, Italy; ^2^Department of Microbiology, Cell and Tumor Biology (MTC), Karolinska Institutet, Stockholm, Sweden; ^3^Department of Molecular Medicine and Medical Biotechnologies Federico II University, Naples, Italy; ^4^Research Center of Advanced Biochemistry and Molecular Biology, Department of Experimental and Clinical Medicine, Magna Græcia University of Catanzaro, Catanzaro, Italy; ^5^Laboratory of Proteomics, Research Center of Advanced Biochemistry and Molecular Biology, Department of Experimental and Clinical Medicine, Magna Græcia University of Catanzaro, Catanzaro, Italy; ^6^Division of Clinical Pathology, Department of Health Sciences, Magna Graecia University, Catanzaro, Italy; ^7^Department of Health Sciences, University of Catanzaro Magna Graecia, Catanzaro, Italy; ^8^Department of Surgery, Massachusetts General Hospital, Harvard Medical School, Boston, MA, United States; ^9^CIS for Genomics and Molecular Pathology, Magna Graecia University of Catanzaro, Catanzaro, Italy

**Keywords:** MHC-I, NK cells, iron, IFNγ, STAT1, HLA

## Abstract

The ability of pathogens to sequester iron from their host cells and proteins affects their virulence. Moreover, iron is required for various innate host defense mechanisms as well as for acquired immune responses. Therefore, intracellular iron concentration may influence the interplay between pathogens and immune system. Here, we investigated whether changes in iron concentrations and intracellular ferritin heavy chain (FTH) abundance may modulate the expression of Major Histocompatibility Complex molecules (MHC), and susceptibility to Natural Killer (NK) cell cytotoxicity. FTH downregulation, either by shRNA transfection or iron chelation, led to MHC surface reduction in primary cancer cells and macrophages. On the contrary, mouse embryonic fibroblasts (MEFs) from NCOA4 null mice accumulated FTH for ferritinophagy impairment and displayed MHC class I cell surface overexpression. Low iron concentration, but not FTH, interfered with IFN-γ receptor signaling, preventing the increase of MHC-class I molecules on the membrane by obstructing STAT1 phosphorylation and nuclear translocation. Finally, iron depletion and FTH downregulation increased the target susceptibility of both primary cancer cells and macrophages to NK cell recognition. In conclusion, the reduction of iron and FTH may influence the expression of MHC class I molecules leading to NK cells activation.

## Introduction

Iron is an important component in the regulation of metabolic homeostasis, but high levels are dangerous since they generate highly toxic reactive oxygen species via the Fenton's reaction. Therefore, the iron concentration must be tightly controlled, both systemically and intracellularly (1).

Iron is of pivotal importance for our immune system and, at the same time, is essential for the survival of several pathogenic microorganisms. Pathogens obtain iron to support their proliferation from host iron containing proteins and the maintenance of low iron concentration in the serum is considered an antibacterial defense (2). Moreover, iron-overloaded hosts are more susceptible to infections including yersinosis, salmonellosis, tuberculosis, and AIDS (3, 4).

Clinical studies in Africa have demonstrated how iron repletion in iron-deficient individuals was able to reactivate pre-existing infections (5).

More recent epidemiological studies indicate that nutritional iron overload can increase mortality from active tuberculosis (6, 7). These observations may be explained by the evidence that increased provision of iron better supports pathogens metabolism but may also reflect a role for iron in modulating both innate and acquired immunity (8, 9).

Recently, abnormal iron metabolism has also been associated with tumorigenic processes (10, 11). Free iron contributes to the formation of reactive oxygen species (ROS) that, in turn, cause oxidative stress, promoting mutagenesis, DNA breaks, oncogene activation, and tumor suppressor genes inhibition (12). Moreover, iron is needed by cancer cells to sustain a high proliferation rate (13).

Since both infective and neoplastic diseases outcome is strongly influenced by the immune system the relationship between iron metabolism and immune response may be important to better understand the key events in evolution of such pathological processes. This is the ambition of the present study in which we focus our attention on MHC class I molecules, which can regulate NK cells, one of the two major cytotoxic lymphocytes populations (NK and CD8^+^T cells), involved both in cancer and infections.

NK cells are large granular lymphocytes and members of the Innate Lymphoid Cells group 1 (ILC1) subset (14). They are potent cytotoxic effectors able to recognize, without prior specific sensitization, bacteria, and virus-infected, transformed, and allogeneic cells while sparing autologous healthy cells (15). This capability depends on the integrated balance of signals generated by activating and inhibitory NK cell receptors that scrutinize the surface of potential target cells. The inhibitory signals are generated by the binding of MHC class I molecules to Killer cell Immunoglobulin-like Receptors (KIRs) and to Immunoglobulin-Like Transcript (ILT, also known as LIR, CD85) receptors in humans, to Ly49 receptors in mice, and to the CD94/NKG2A heterodimer (16, 17) in both species. On the other hand, NK cells activation depends on receptors such as NK group 2 member D (NKG2D), Natural Cytotoxicity Receptors (NCRs) (18), 2B4 and DNAX accessory molecule-1 (DNAM-1) (19). Increased NK susceptibility therefore results from the increased expression of activating ligands, decreased expression of MHC class I molecules or other inhibitory ligands, or a combination of these two events (20).

Several studies show a functional connection between iron metabolism and MHC antigens. The most frequently mutated gene in patients affected by Hereditary Hemochromatosis (HH), a heterogeneous inherited disorder associated to mutations in genes involved in Hepcidin production characterized by iron overload and tissue damage, is Hfe (21, 22). Hfe encodes a non-classical, beta 2 microglobulin (β2m)-associated MHC class I molecule and is localized 4 Mb telomeric to HLA-A2. In the liver, Hfe protein promotes signal transduction from TFR2 receptor when transferrin saturation increases and this activates hepcidin production to reduce iron absorption and recycle (23). The Hfe gene is in linkage disequilibrium with an ancestral haplotype carrying the HLA antigens A^*^03 and B^*^07. Individuals with two copies of this haplotype show a more severe iron overload phenotype (24).

It has been shown that “double knockout” mice for Hfe and the β2m genes accumulate more tissue iron in comparison with what observed in mice with single knockout for Hfe gene (25). Thus, de Sousa et al. (26) found increased hepatic iron concentration in mice lacking classical MHC class I molecules. Moreover, the mycobacteria burden, which is strongly influenced by iron availability, is elevated in mice lacking β2m (27) as well as in mice fully deficient for MHC class I molecules (28). Notably, these mice strains have a hereditary iron-overload phenotype (26).

Beyond the regulation of its uptake, intracellular iron concentration is maintained stable by buffering iron excess in Ferritin. Ferritin is composed by 24 subunits of acidic/heavy (H) (FTH) and basic/light (L) (FTL) chains. It acts as intracellular iron nanocage that accumulates large amounts of iron in its cavity (up to 4,000 per molecule) to prevent oxidative stress, and to release it in iron deprivation conditions (29). NCOA4 protein controls the iron-dependent recycling of ferritin iron, in a process named “ferritinophagy.” It was shown that under conditions of low iron, NCOA4 binds ferritin and targets its degradation to recycle iron, while in high iron conditions, NCOA4 is ubiquitinated and degraded by proteasome, favoring iron sequestration (30).

Here, we have investigated the role of iron and its binding protein FTH in modulating MHC class I expression and NK cell recognition. The study was first performed in mice and in human using an *in vitro* experimental setting. The results were validated *ex vivo* in NCOA4-null mice.

## Materials and Methods

### Cell Culture

MM07m (supraclavicular lymph node metastasis), MM07m shFTH (FTH-silenced) cells were cultured in RPMI 1640 (Life Technologies, Monza, Italy) supplemented with 10% FBS, 10 units/ml penicillin, and 10 mg/ml streptomycin. MCF-7 and MCF-7 shFTH cells were cultured in Dulbecco's modified Eagle's medium (Life Technologies, Monza, Italy) supplemented with 10% FBS, 10 units/ml penicillin, and 10 mg/ml streptomycin. Cells were grown at 37°C in a 5% CO_2_ atmosphere. Freshly explanted melanoma cell lines were obtained from patients after informed consent, according to previously described procedure (31) at the Fondazione IRCCS Istituto Nazionale dei Tumori, Milan, Italy. The cells derived from the patients were named Mel-30 and Mel-35. Cells were cultured in RPMI 1640 medium supplemented with 10% heat inactivated fetal calf serum (FCS), 10 units/ml penicillin and 10 mg/ml streptomycin and passaged every 2–3 days.

### Preparation of Lentiviral Supernatants and Transduction of MM07_m_ and MCF7 Cells

Lentiviral preparations and transductions were performed as previously described (32, 33). The supernatants were used to cross-transduce MM07m and MCF-7 cells in the presence of 8 μg/ml polybrene (Sigma-Aldrich, Saint Louis, Missouri, United States) and positive clones were isolated by puromycin selection (1 μg/ml).

### NK Cell Generation Assay

NK cells preparation was done as described elsewhere (34). Briefly, peripheral blood mononuclear cells (PBMCs) were isolated from buffy coats of healthy donors and from four hemochromatosis patients by Biocoll Separating Solution (Biochrom GmbH, Berlin, Germany) density gradient centrifugation. Enriched NK cells were isolated from the separated PBMCs utilizing the NK cell isolation kit and VarioMACS (Miltenyi Biotec, Bologna, Italy) according to the manufacturer's instructions. The purity of the isolated CD3^**−**^CD56^+^ NK cell populations was ≥95%. Freshly enriched NK cells were suspended in RPMI 1640 culture medium (Life Technologies, Carlsbad, California) supplemented with penicillin (100 IU/ml) and streptomycin (100 mg/ml), and 10% FBS. In the cytotoxicity with K562 cells, NK cells were treated with 100 μM of Deferoxamine (DFO) for 16 h. After that cells were processed for the cytotoxicity assay experiments as described below.

### Cytotoxicity Assay

Cytotoxicity was measured using the fluorescent 5,6-carboxyfluorescein diacetate (CFDA) NK assay or using a standard 4-h [^51^Cr]-release assay (35). In CFDA NK assays, cytotoxicity was analyzed by flow cytometry. Briefly, the target cells were labeled with CFDA-mixed isomers (Invitrogen, Milan, Italy). Target cells were mixed with effector cells at different Effector: Target ratios (E:T). The incubation was performed in 96-well U-bottom plates at 37°C in a humidified 5% CO_2_ incubator for 3 h. The specific lysis of target cells was calculated as follows: % specific lysis = (CT–TE/CT) × 100, where CT indicates mean number of fluorescent target cells in control tubes and TE indicates mean number of fluorescent cells in target plus effector tubes.

### Antibodies and FACS Analysis

The cell lines were analyzed by immunofluorescence and flow cytometry analysis using the following Abs: W6/32 (anti-HLA class I PE conjugated), HLA-E (clone 3D12 PE conjugated), HLA-G (clone 87G PE conjugated), CD86 (clone GL-1 PerCP/Cy5.5 conjugated), and CD14 (clone M5E2 APC conjugated) (BioLegend). Cells were incubated with heat inactivated human serum for 15 min and isotype-matched controls were used to set up the negative values. Mouse splenocytes were stained with fluorescently conjugated anti-mouse antibodies: NK1.1 (PK136), CD3 (145.2C11), Ly49A (YE1/48.10.6), KLRG1 (2F1), NKp46 (29A1.4), DNAM-1 (10E5), CD11b (M1/70), CD27 (LG.3A10), LFA-1 (H155-78),2B4 (2B4), (Biolegend); Ly49G2 (4D11), H2-Db (KH95), H2-Kb (AF6-88.5) and NKG2D (CX5) (BD Biosciences); Ly49I (YLI-90); and NKG2A (20d5) (eBioscience). Ly49C (4LO) hybridoma was a kind gift from Suzanne Lemieux (INRS–Institut Armand-Frappier, Laval, Quebec). Dead cells were excluded using Live/Dead™ Fixable Aqua Dead Cell Stain Kit, for 405 nm excitation (ThermoFisher). Samples were analyzed by a BD FACScan™ (Becton Dickinson, Mountain View, CA) or BD FACSCanto™ II. For human cell lines and murine splenocytes, cells were first gated on physical parameters (SSC-H vs. FSC-H) and then for singlets (FSC-A vs. FSC-H). For purified human macrophages cells were first gated on physical parameters (SSC-H vs. FSC-H), then for singlets (FSC-A vs. FSC-H), and the CD14 positive population was further analyzed. Analyses were performed by using FlowJo software (TreeStar Inc. Ashland, OR, USA).

### Flow Cytometry Measurements of Labile Iron Pool (LIP). Calcein Test

HeLa shCTRL and shFTH clones were loaded with 0.25 μM calcein acetoxymethyl ester (CA-AM) (Sigma 56496) for 15 min, then washed with PBS1X and treated or not for 1 h with the iron chelator DFO (500 μM). The fluorescence (FL1-H) of Calcein-stained cells were analyzed by flow cytometry and the difference in the Mean Fluorescence Intensity (MFI) between DFO-treated and untreated cells was used to calculate the amount of Labile Iron Pool (LIP).

### Protein Studies

HeLa shCTRL and shFTH were lysed in a buffer containing 50 mM N-2-hydroxyethylpiperazine-N'-2-ethanesulfonic acid (HEPES; pH 7.5), 1% (vol/vol) Triton X-100, 150 mM NaCl, 5 mM EGTA, 50 mM NaF, 20 mM sodium pyrophosphate, 1 mM sodium vanadate. Protein extracts (50 μg) were diluted in Laemmli sample buffer and subjected to SDS-PAGE and by standard Western blot techniques using the following antibodies: anti FTH1 (D1D4) (Cell Signaling) (1:1000) and anti Phospho-Stat1 (Tyr701) (Cell Signaling) (1:1000); anti Stat1 p84/p91 (C-136) sc-464 (1:500) (Santa Cruz); Anti-HLA Class I ABC antibody [EMR8-5] (ab70328) (1:1000) (Abcam).

### Splenocytes Isolation From NCOA4-Null Mice

NCOA4 WT and KO in C57BL6 genetic background mice were maintained under specific pathogen-free conditions in the animal facility of the Department of Molecular Medicine and Medical Biotechnologies Federico II University, Naples, Italy. This study was carried out in accordance with the principles of the Basel Declaration and recommendations of Italian regulations for experiments involving animals (D.L. 26/2014). The protocol was approved by the Italian Ministry of Health (authorization 774/2015PR). Genetic screening of mice and MEFs isolation were conducted by genomic PCR as described elsewhere (36).

Spleens were mechanically disrupted into single cell suspension and erythrocytes were eliminated by incubation for 2 min in RBC lysis buffer (55 mM NH_4_Cl, 12 mM NaHCO_3_, 0.1 mM EDTA) on ice. Splenocytes were then washed two times in PBS and treated separately for RNA or protein extraction.

### RNA Isolation and Real Time q-PCR Analysis

Total RNA was extracted using the TRIzol method (Invitrogen Life Technologies) according to the manufacturer's instructions and reverse-transcription was subsequently performed with a reverse transcription kit (Invitrogen).

Each fragment was amplified in a 20 μl reaction containing 1X SYBR Green I PCR Master mix (Bio-Rad Laboratories), 20 pmol of each primer pair, 20 ng of cDNA (total RNA equivalent), and nuclease-free water. The thermal profile consisted of 1 cycle at 95°C for 3 min followed by 45 cycles at 95°C for 10 s, 60°C for 10 s, 72°C for 20 s. The PCR reaction was followed by a melting profile analysis. A glyceraldehyde 3-phosphate dehydrogenase (GAPDH) cDNA fragment was amplified as the internal control for the amount of cDNA in the qPCR. Each experiment was performed in triplicate.

The primers sequences were as follows for human samples and for murine samples:

Human samples:

FTH for 5′-CATCAACCGCCAGATCAAC-3′

FTH rev 5′-GATGGCTTTCACCTGCTCAT-3′

GAPDH for 5′-TGATGACATCAAGAAGGTGGTTGAAG-3′

GAPDH rev 5′-TCCTTGGAGGCCATGTGGGCCAT-3′

HLA-I for 5′-CCTTGTGTGGGACTGAGAGG-3′

HLA-I rev 5′-CAGAGATGGAGACACCTC-3′

Mouse samples:

GAPDH for 5′-AACACCACCATGGAGAAGGC-3′

GAPDH rev 5′-ACAGCCTTGGCAGCACCAGT-3′

FTH for 5′-CATCAACCGCCAGATCAAC-3′

FTH rev 5′-GATGGCTTTCACCTGCTCAT-3′

FTL for 5′-CTTGCCCGGGACTTAGAGCA-3′

FTL rev 5′-ATGGCTGATCCGGAGTAGGA-3′

### Macrophages Cell Generation and Treatment With DFO

Primary human macrophages where isolated from PBMCs by adherence. First, white blood cells where isolated from healthy donor buffy coats by Ficoll-Paque™ Plus solution (GE Healthcare Bio-science AB) density gradient centrifugation at 2,200 rpm for 30 min. PBMCs were suspended in RPMI 1640 medium supplemented with 10% autologous serum and 100 U/ml penicillin and 100 μg/ml streptomycin and cultured in 75 cm^2^ plastic flask at 37°C, 5% CO_2_. After overnight culture, the medium containing the non-adherent cells was decanted and replaced with fresh medium with or without DFO (Sigma Aldrich) at a final concentration of 100 μM for additional 16 h. After the treatments, the adherent macrophages were gently scraped with a plastic cell scraper.

### Statistical Analysis

Results of experimental points obtained from multiple experiments were reported as mean ± SD. GraphPad Prism version 7 was applied for data analysis. Significance levels were determined by a paired Student's *t*-test analysis or repeated measured ANOVA based on the dataset that were compared to each other. *P* ≤ 0.05 was considered significant. Column statistic one-sample *t*-test was used to perform the analysis of samples referred as % of control by setting the hypothetical value to 100. In all the figures, ^*^p ≤ 0.05, ^**^p ≤ 0.01, ^***^p ≤ 0.001.

## Results

### Ferritin Heavy Chain (FTH) Abundance Modulates Cell Surface MHC Expression

**Figure 1:**
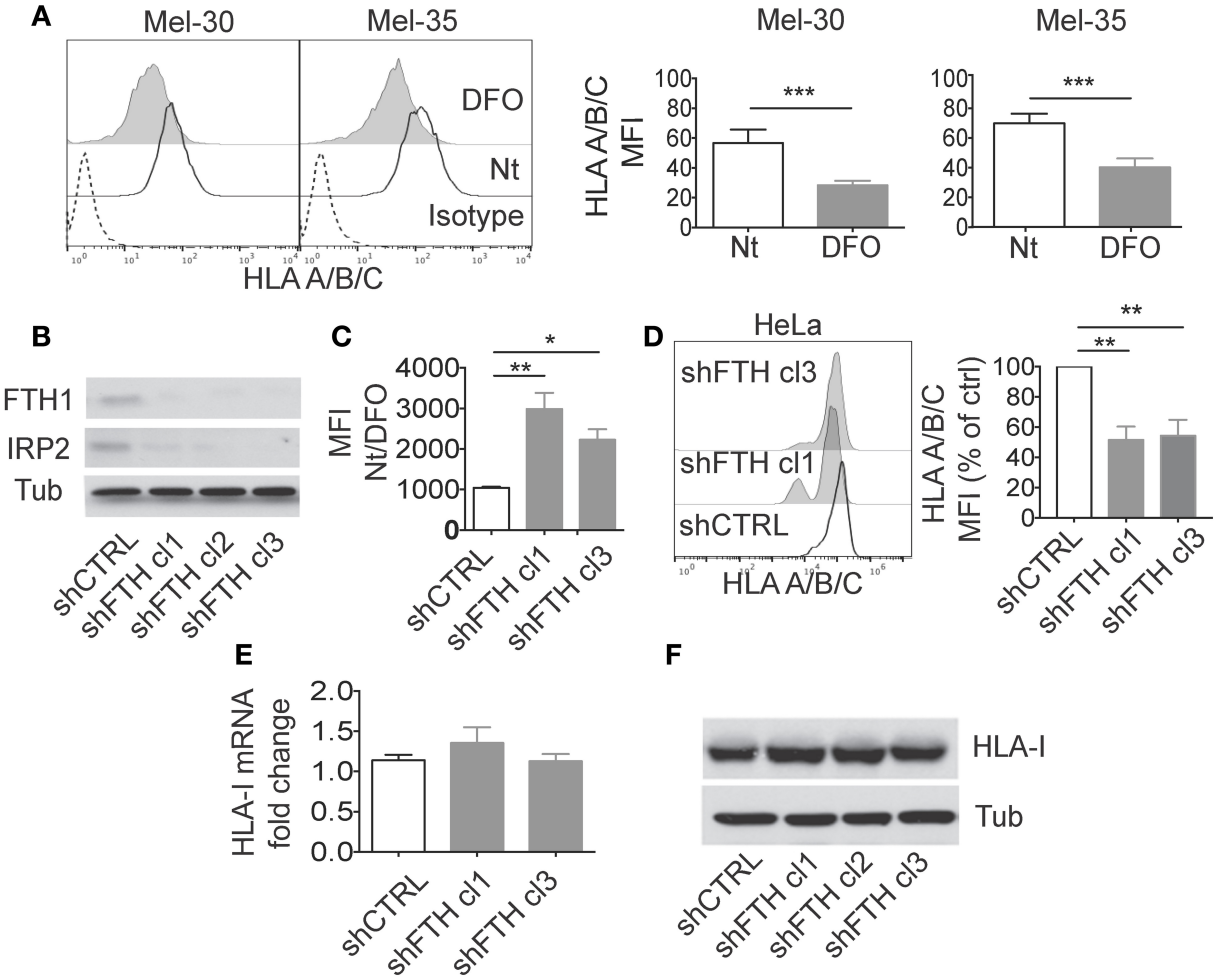
Ferritin Heavy Chain (FHC) modulates the expression of MHC-class I molecules. **(A)** Mel30 and Mel35 primary melanoma cells where treated overnight with DFO and classical (HLA A/B/C) MHC class I surface expression was measured by flow cytometry. The dashed curve in the two histograms represents the isotype control; the white curve represents the untreated (Nt) control cells and the filled gray curve represents cells treated with DFO. Columns show statistical analysis of three consecutive independent experiments. *P*-values were calculated using paired Student *t-*test (^*^p < 0.05; ^**^p < 0.01; ^***^p < 0.001). **(B)** Western blotting analysis of Ferritin Heavy Chain (FTH1) and Iron binding protein 2 (IRP2) in HeLa cell lines stably transfected with shCTRL or shFTH. Three different clones were selected with puromycin (2.5 ug/ml). Tubulin was used as loading control. **(C)** Flow cytometry measurements of labile iron pool (LIP) in HeLa shCTRL and two different shFTH clones. Cells were treated with Calcein-AM, followed by 1 h incubation with or without DFO. The ratio in the mean fluorescence intensity (MFI) between DFO-treated and untreated cells of three different experiment was measured and is presented in the histogram. *P*-values were calculated using 2-tailed Unpaired Student *t*-test. ^*^p < 0.05, ^**^p < 0.01. **(D)** Representative Flow cytometry analysis of classical (HLA A/B/C) surface expression in HeLa shCTRL and two different shFTH clones. **(E)** Relative expression of MHC-I mRNA in HeLa shCTRL and two different shFTH clones with respect to the housekeeping gene GAPDH. **(F)** Western blotting analysis of MHC-I **(A–C)** in HeLa cells stably transfected shCTR or shFTH.

To investigate whether iron metabolism can directly modulate MHC class I expression interfering with immune responses (and NK activity), we modulated iron levels and evaluated MHC class I expression in different cellular systems. Primary human melanoma cells (Mel-30 and Mel-35) were cultured in iron-depleted conditions by treatment with the iron chelator Deferoxamine (DFO) (Figure 1A). DFO led to a down regulation of MHC-class I expression on cell surface, including the non-classical HLA-E molecule (Figure 1A; Supplementary Figure 1A). We observed a reduction of MHC molecules also on primary macrophages cultured in the presence of DFO. In this case, the effect occurred on the non-classical MHC-class I molecules (HLA-E), but not on classical ones as shown in Supplementary Figure 1B. Interestingly we also observed a reduction of both MHC-class II and CD86 surface expression after DFO treatment. The surface molecule CD14 was not affected (Supplementary Figure 2).

Iron chelation by DFO reduces labile iron pool (LIP) concentration and promotes ferritin degradation by autophagy to mobilize intracellular iron deposits. In order to address whether DFO-dependent modulation of MHC expression was due to reduction of LIP or to reduction of intracellular ferritin, we tested MHC expression in HeLa cells in which ferritin abundance was reduced by shRNA. Upon stable transfection with shRNA for the FTH, we generated several clones of HeLa cell line which display marked reduction of ferritin, measured by western blotting (Figure 1B), and increase of LIP, measured by Calcein assay (Figure 1C). In HeLa shFTH cells, reduction of ferritin by itself induced a decrease of MHC-class I expression on cell surface, albeit iron concentration was increased, indicating that DFO effects were due to reduction of ferritin rather than reduction of intracellular iron concentration (Figure 1D). Decreased expression of MHC class I molecules was also observed in MM07m and MCF7 cell lines in which ferritin protein abundance was reduced by shRNA (Supplementary Figures 1C,D). Similar results were obtained using two different shRNA for FTH (data not shown).

Interestingly, in HeLa shFTH cells decreased expression of MHC class I molecules on cell surface was not due to decreased transcription, as shown by rtPCR of MHC class I mRNA levels (Figure 1E), or decreased overall translation of such antigens, as shown by western blotting of total cellular proteins (Figure 1F). These results suggest that decreased ferritin levels were likely affecting MHC protein maturation and transport to cell surface rather than total protein expression.

To corroborate these observations, we used a reciprocal approach, asking whether the increase of intracellular ferritin could lead to augmentation of MHC class I expression. To this aim we analyzed in an *in vivo* setting represented by NCOA4 KO mice, in which impairment of ferritinophagy leads to ferritin accumulation in cells and tissues (37).

**Figure 2:**
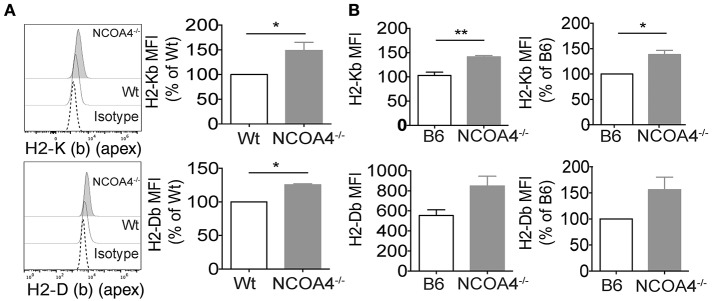
*In vitro* and *ex vivo* modulation of ferritin heavy chain and MHC-I expression in mouse. **(A)** Immortalized Embryonic fibroblasts derived from NCOA4 WT and KO mice (MEFs) were surface stained for MHC class I molecules H2 K (b) (apex) and H2 D (b) (apex) and analyzed by flow cytometry. Data are representative of three independent experiments. **(B)** Cell surface levels of H2-K (b) (apex) and H2-D (b) (apex) were analyzed on freshly explanted splenocytes of NCOA4 KO mice compared to those of wt mice. White column represents wt (B6) mice while gray column corresponds to NCOA4 KO mice. Data are representative of three consecutive experiments. *P*-values were calculated using 2-tailed Unpaired Student *t-*test. ^*^p < 0.05; ^** ^*p* < 0.01; ^***^ *p* < 0.001.

MHC class I molecules expression levels were augmented on the cell surface of *in vitro* grown NCOA4 KO mouse embryonal fibroblasts (MEFs) compared to MEFs from wt animals (Figure 2A). Furthermore, cell surface levels of K (b) (apex) and H2-D (b) (apex) were significantly higher on freshly explanted splenocytes of NCOA4 KO mice compared to those of wt mice (Figure 2B).

Overall these data strongly support the observation that intracellular ferritin levels affect the abundance of MHC class I antigens on cell surface, possibly affecting innate and adaptive immune response.

### Interferon-Gamma Induced MHC Class I Molecules Expression Is Affected by Iron Levels

IFN-γ induces maturation of macrophages, and it is a key cytokine for optimization of antigen presentation in infections, by virtue of its capacity to induce high expression of MHC molecules. Given our observation that iron and ferritin levels can impinge on MHC expression, we set to analyze the MHC class I regulation by IFN-γ either in low iron conditions (DFO treatment), or by interference of FTH expression.

**Figure 3:**
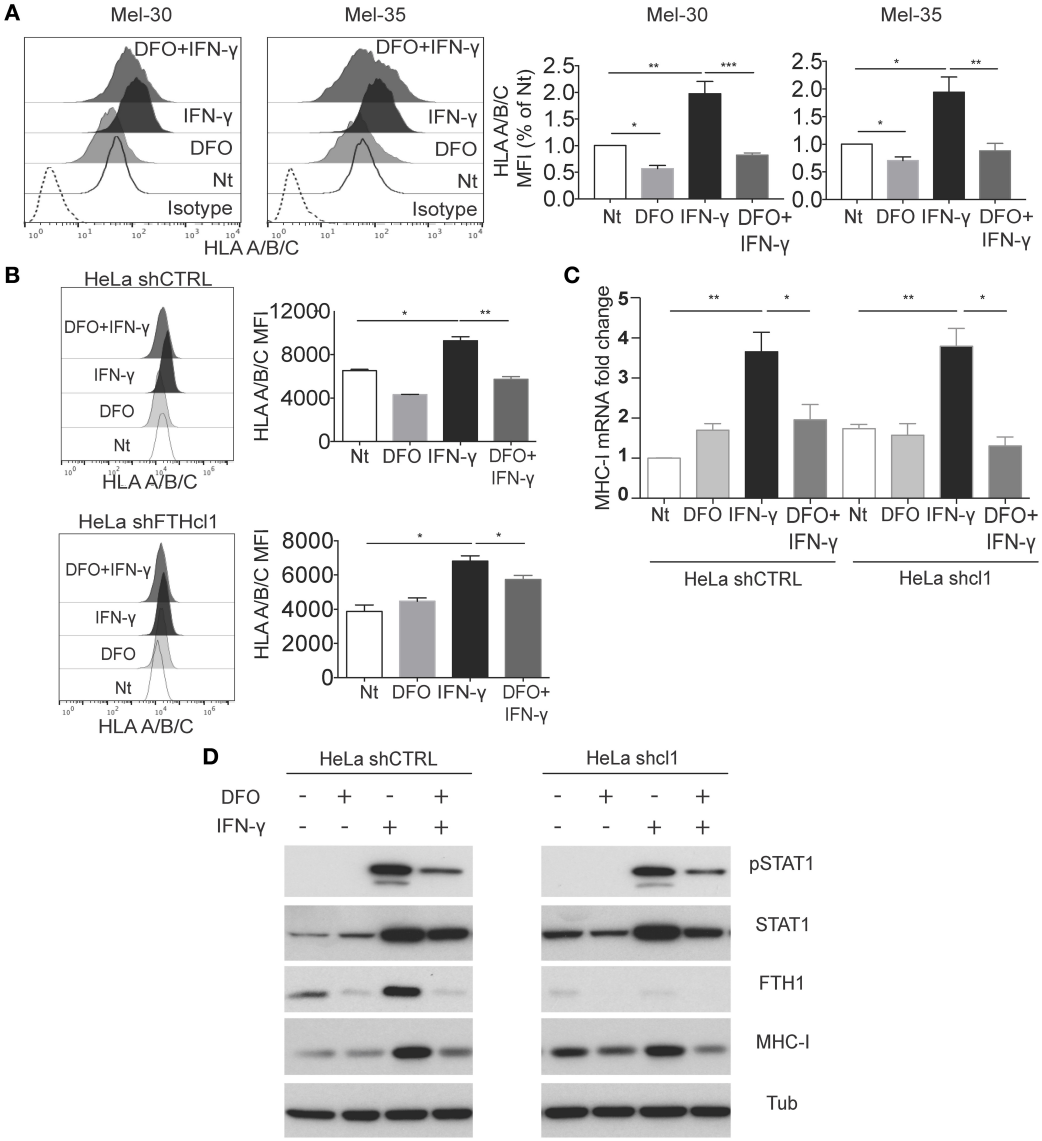
Interferon-γ stimulation is affected by iron levels. **(A)** Mel30 and Mel-35 primary melanoma cell were grown in presence of DFO, IFN-γ, or a combination of both. Cells were stained for MHC class I molecule (HLA-A/B/C), and analyzed by flow cytometry. The dashed curve in the histograms represents the isotype control; the white curve represents the untreated control cells; the light gray curve represents the treatment with DFO; the black curve represents cells stimulated with IFN-γ, and the dark gray curve represents cells treated with DFO + IFN-γ. Columns show statistical analysis of three independent experiments. Statistical analysis was performed by ANOVA followed by Holm-Sidak's multiple comparisons test. ^*^p < 0.05; ^**^p < 0.01; ^***^p < 0.001. **(B)** Representative flow cytometry analysis of classical (HLA-A/B/C) surface expression in HeLa shCTR and shFTH clone 1, grown for 12 h with or without DFO (100 uM) and then stimulated for 24 h with IFN-γ (20 ng/ml). Statistical analysis was performed using Student *t*-test comparing untreated to IFN- γ-treated cells, and comparing IFN-γ to IFN-γ /DFO treated cells. ^*^p < 0.05, ^**^p < 0.01 **(C)**. HeLa shCTR and shFTH clone 1 were treated as described in Figure 3B, then total RNA was extracted, retro-transcribed into cDNA and the expression of MHC-I mRNA was analyzed compared to the housekeeping gene GAPDH. Statistical analysis was performed using Student's *t*-test comparing untreated to IFN-γ-treated cells. **(D)** Western blotting analysis of the indicated antibodies in HeLa shCTR and HeLa shFTH clone 1 cells treated as in Figure 3B. pSTAT1 (Y701) antibody was used to monitor STAT1 activity and tubulin was used as loading control.

When the primary melanoma cells, Mel-30 and Mel-35, were grown in presence of DFO, concomitant stimulation with IFN-γ failed to increase the classic MHC class I expression (Figure 3A). DFO treatment also prevented the effect of the IFN-γ on expression of HLA-E (Supplementary Figures 3A,B). This was a specific effect on MHC class I molecules since the DFO treatment was able to induce (rather than decrease) a different cellular surface marker, CD155, expression both at the steady state and upon IFN- γ treatment (Supplementary Figure 3C).

These data led to the question of how low iron and/or, possibly, decreased FTH levels induced by DFO treatment, can interfere with the IFN-γ-induced MHC expression. To address this question, we analyzed how HeLa cells, either treated with DFO or silenced for FTH (shFTH), responded to IFN-γ stimulation. In HeLa shFTH, although cell surface HLA levels were lower compared to shCTRL cells, as previously described (Figure 1), HLA expression on cell surface increased upon IFN-γ treatment (Figure 3B). On the contrary, DFO treatment hindered MHC class I molecules expression on cell surface upon IFN-γ stimulation both in HeLa shCTRL and shFTH cells, indicating that it is low iron concentration (LIP), rather than low ferritin, to be responsible for blockade of IFN-γ-induced MHC expression (Figure 3B). In order to understand how DFO (i.e., low LIP) was obstructing IFN-γ induction of MHC expression, we analyzed MHC class I mRNA, and total protein levels in both HeLa shCTRL and shFTH cells. As shown in Figures 3C,D, treatment with DFO blocked mRNA induction and protein accumulation upon IFN-γ treatment in both cell types, while FTH silencing, *per se*, did not affect IFN-γ stimulatory effects, as expected.

Since one of the major signaling events acting downstream IFN-γ receptor is STAT1 transcription factor phosphorylation, nuclear translocation, and gene transcription activation, we tested whether DFO was able to directly interfere with IFN-γ-induced STAT1 activation. As shown in Figure 3D, DFO treatment markedly reduced STAT1 phosphorylation upon IFN-γ stimulation in shCTRL and HeLa shFTH cells, indicating that LIP reduction directly attenuates IFN-γ receptor signaling. In accordance to what previously shown, FTH silencing did not affect IFN-γ-induced STAT1 phosphorylation. Over all these data suggest that labile iron acts directly on IFN-γ signaling by obstructing STAT1 activation, while intracellular ferritin abundance does not, only impinging MHC class I expression on the cell surface, as shown already in unstimulated cells (Figure 1).

### Reduced Iron Concentration and Ferritin Heavy Chain Is Associated With NK Cell Susceptibility of Established Tumor Cell Lines and Primary Melanoma Cells

**Figure 4:**
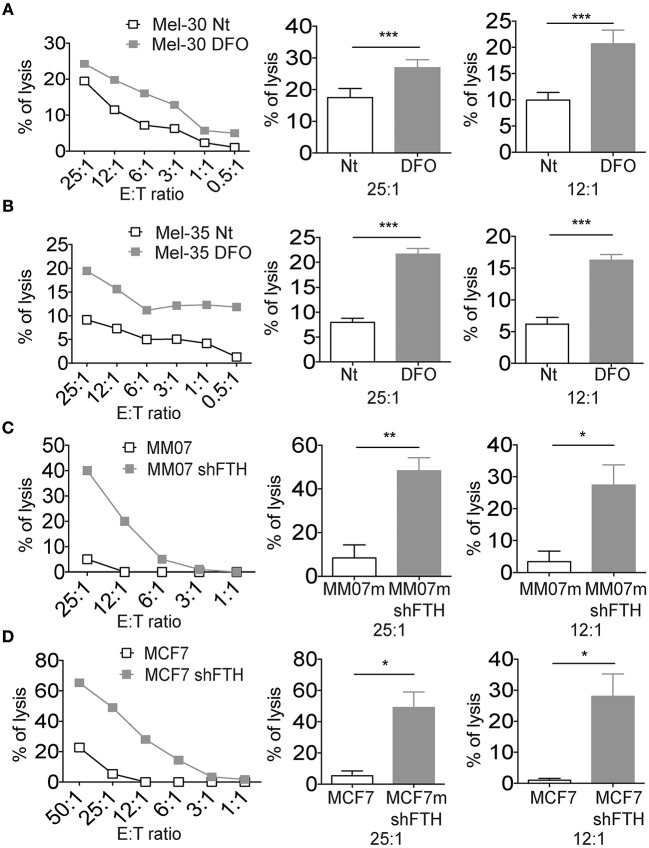
Iron and Ferritin Heavy Chain reduction is associated with NK cell susceptibility of established tumor cell lines and primary melanoma cells. **(A,B)** Representative cytotoxicity experiments in which two primary melanoma cell lines, Mel-30 and Mel-35, where treated overnight with DFO. Curves show the NK cell recognition of DFO treated cells (gray squares), and untreated cells (white squares). Columns show statistical analysis of three consecutive experiments at the effector:target ratio of 25:1 and 12:1, respectively. **(C,D)** Representative cytotoxicity experiments in which MM07m shFTH and MCF7 shFTH (gray squares), or scramble (white squares) were tested for their susceptibility to NK cell killing. Column show statistical analysis of five consecutive experiments at the effector:target ratio of 25:1 and 12:1, respectively. *P*-values were calculated using paired Student *t-*test (^*^p < 0.05; ^**^p < 0.01; ^***^p < 0.001).

Since iron concentration (and consequently FTH levels) can be reduced in the very early stages of the infection, due to iron consumption or decreased uptake, we reasoned that innate immunity might be able to sense such iron fluctuation via changes in MHC expression in target cells expressing reduced levels of FTH. To experimentally challenge this hypothesis two melanoma cell lines were grown in low iron concentration medium and then used separately as target cells in cytotoxicity assays with NK cells (Figures 4A,B). Target cells became more susceptible to NK cell killing in the presence of DFO in the medium. Since we demonstrated that it is ferritin abundance rather than LIP that regulates MHC expression on cell surface (Figure 1), we determined whether the specific silencing of FTH in tumor cell lines would result in increased NK cells killing activity. The results indicated that shFTH transfection in tumor cell lines triggered a more robust NK cell recognition compared to the activity against mock transfectants cells (Figures 4C,D). Similar results were obtained with primary macrophages as target cells (Supplementary Figure 4A).

In order to exclude that DFO affects NK cells directly, irrespective of its effects on MHC expression of target cells, freshly isolated NK cells were treated with DFO and used in a cytotoxicity assay using MHC negative K562 as target cells. As shown in Supplementary Figure 4B, no change in the tumor cells killing was observed, arguing against the possibility that the iron levels directly modulate NK cell function.

We also measured the surface expression levels of activating NK cell ligands on MM07m cells silenced or not for FTH. The reduction of FTH by shRNA led to an increase of DNAM1 ligands CD155 (PVR) and CD112 (NECTIN2) on the surface of MM07m cells (Supplementary Figure 4C). While we did not observe any modulation of NKG2D ligands (Supplementary Figure 4D).

Thus, the iron/ferritin down modulation leads to a highly NK cell susceptible immune phenotype with low surface levels of MHC class I inhibitory molecules and increased amount of membrane associated activating DNAM-I ligands.

## Discussion

Our study unveiled a novel cross talk between iron metabolism and MHC molecules.

Decreased MHC class I expression on the cell surface was observed upon DFO treatment, which by chelating iron induces ferritin degradation as well as shRNA for FTH (Figure 1). Vice versa, increased MHC class I surface expression was observed in mouse model of ferritin accumulation such as NCOA4 KO mice that show impaired ferritin degradation (Figure 2).

The data obtained both *in vitro* and *ex vivo* clearly demonstrated that it is not DFO-induced low iron *per se* to promote reduction of MHC class I expression but rather DFO-induced decrease of ferritin abundance. Thus, cells silenced for FTH display increased LIP but low levels of MHC (Figure 1) while NCOA4 null cells display low or normal LIP but high levels of ferritin and MHC class I proteins (Figure 2 and data not shown). It should be stressed that, rather than promoting a reduction in MHC class I mRNA or total protein abundance, DFO or shRNA FTH induced a reduction of the abundance of MHC on cell surface (Figure 1), indicating an impairment of posttranslational maturation that we sought to investigate in more detail in the future. It is also important to underline that MHC Class I antigen mRNAs do not contain Iron Responsive Elements (IREs) and are not known to be modulated by Iron Regulatory Proteins (IRPs).

Of note, downregulation of classical MHC class I (either by DFO or by shRNA FTH) was observed in cancer cells but not in macrophages while the non-classical MHC class I were reduced in both (Supplementary Figure 1). Several explanations for classical MHC class I molecules could account on the different behavior in such cellular settings. First of all, the baseline membrane associated levels of MHC class I molecules on the tumor cells surface are two logs lower than that measured on macrophages cells, making more difficult to observe with a pan MHC class I antibody the potential fluctuation of single allele product. Indeed, it is well-known that tumor cells have several concomitant operating mechanisms to reduce their MHC class I expression on cell membrane. In addition, the tumor associated neo-antigens may have low affinity for the MHC class I/β2m heterodimers making them more instable and consequently easier to down regulate. While in our study we analyzed mainly MHC class I and non-classical MHC molecules as HLA-E, we cannot exclude that other genes associated with MHC expression like TAP1 and TAP2 or other antigen processing machinery related genes could be regulated as well.

MHC class I molecules are expressed and present peptides on almost all nucleated cells of the body. Maturation and correct folding of MHC class I is a multistep process, where the heavy chain, β2-microglobulin, and the peptide are all essential for surface expression of the complex. In ER heavy chains first bind to the chaperones immunoglobulin heavy chain-binding protein (BiP) and calnexin, which is then replaced by calreticulin upon binding to β2m. The heavy chain-β2m molecule associates with MHC class I loading complex (LC) which consists of several ER-resident proteins including transporter associated with antigen processing (TAP). The peptides are generated in the cytosol by the proteasome, transported into the ER lumen by TAP, and then further trimmed by ER-resident aminopeptidase. Correct folding, association, and loading depend heavily on glycosylation of the heavy chain. Sequential interaction with a set of dedicated proteins guides transport of the assembled complex to the cell surface.

Interestingly, levels of both BiP and calreticulin were increased in iron-burdened astrocytoma cells (38), and BiP expression was increased in dietary iron-loaded mice (39). Change in ER-resident chaperones levels by iron concentrations could potentially explain both the observed decrease in surface MHC class I, but could also lead to upregulation of other surface molecules that depend on proper protein folding in the ER lumen. To this end, we have observed an increase in the CD112 and CD155, both ligands for the receptors DNAM-1 and TIGIT.

Macrophages play a critical function in iron homeostasis by recycling iron from red cells and storing it in ferritin. These cells use MHC class I and MHC class II to present antigens to the lymphocytes (Antigen Presenting Cells). In tumors, M2-oriented macrophages express low levels of FTH, to release iron to sustain cell proliferation (40, 41).

It has been shown that direct exposure to FTH recombinant protein increases INF-γ production through a direct effect on CD8^+^ T cells and IL-10 production on CD4^+^ T cells indirectly, involving DCs (42). The INF-γ production increases MHC class II (43), PD-L1, and CD86 on APCs (42). We observed a reciprocal regulatory pattern when FTH expression was reduced by exposing the APCs to the iron chelator DFO. The exposure to DFO in APC correlated with reduced expression of MHC class II and CD86 (Supplementary Figure 2). Taking together previous published (42) and our present data it is conceivable that FTH can act as immunomodulatory molecule.

Moreover, our data suggest that the reduction of FTH levels, by DFO leads to macrophages polarization toward M2 like phenotype, characterized by low MHC class II, and CD86 expression (40). The environmental iron concentrations and the related FTH levels is likely critical in the NK cells/M2 cross-talk. Thus, it is conceivable that a reduced uptake of iron may result in a reduced production of FTH that, in turn, will make M2 macrophages more susceptible to NK cells killing.

NK cells are at the interface between innate and adaptive immunity and their activation could alert the adaptive immune response. The NK cells activation is regulated by an integrative balance between activating and inhibitory receptors we demonstrate that iron/ferritin modulation reduce the surface expression of inhibitory MHC class I molecules while have reciprocal effect on the activating DNAM-I ligands. It is conceivable that this provide a mechanistic explanation to the higher NK cell mediated cytotoxic susceptibility of either DFO treated or FTH silenced target cells.

It was already known that iron concentration may modulate NK cell cytotoxic activity (44, 45). Indeed, it might be possible that in the case of an infective disease ferritin could induce NK cells to produce pro-inflammatory cytokines that may contribute to drive the adaptive immunity toward a Th2 immune response, helpful to fight bacterial infections. Low FTH with low HLA-E and classical MHC class I cell membrane levels leads to the NK cells activation (Figure 4). Thus, the acute reduction of ferritin and the consequent reduction of HLA-E may remove the CD94/NKG2A mediated inhibition and permit the fully activation of NK cells. We found conceivable to predict that the NK cell subsets able to recognize monocytes exposed to iron deprivation thus expressing low HLA-E levels are the CD56^bright^ NK cells that are controlled mainly by CD94/NKG2A inhibitory receptors. This NK cell population it is known to be a crucial cytokines producer at the early stages of the infection.

The iron-related immune modulatory mechanism emerging from our data involves also the modulation of MHC class I molecules by IFN-γ (Figure 3). In this case, the low iron concentration, but not the low ferritin, hinders STAT1 phosphorylation and nuclear translocation upon IFN-γ stimulation. Interestingly, a previous report has demonstrated that low iron can promote reduction of IFN-γ receptors and resistance to cytokine stimulation (46). The lack of effect of IFN-γ can contribute to the Th2 skewing of the anti-bacterial immune response as well.

Historically the measurement of the reduction of iron blood concentration has been used in the clinic to monitor the course of an infection. Thus, iron availability plays a pivotal role for bacteria metabolism and virulence. This means that while bacteria use the host iron they will set an ideal condition for NK cells activation due to decrease levels of MHC class I. Thus, it appears conceivable that immune genes regulating the immune response have evolved in order to be able to respond to iron/ferritin decrease induced by infections.

Another intriguing scenario that should be taken in consideration is the role played by iron concentrations in the tumor microenvironment. Correlation between cancer growth and iron/ferritin availability has been reported (47, 48). The hypothesis of a relationship between ferritin and cancer is in line with the reported correlation between FTH and immunomodulatory networks found in Triple Negative Breast Cancer (TNBC) patients (49). Using a combined transcriptomic and proteomic approach it has been shown that the protein levels of the antigen processing genes pathways were significantly associated with the FTH expression and the good prognosis in TNBC patients. Furthermore, among the different factors in antigen processing pathway, the MHC class I proteins showed the strongest association to FTH expression (48).

Finally, we propose a role for FTH and iron metabolism in regulating the recognition of immune molecules of normal and cancerous mammalian cells thus providing a new, and interesting explanation on how the availability of iron can be exploited to modulate our immune system in the battle against microorganisms and cancer. The molecular mechanisms behind the described correlation between FTH and MHC proteins need to be further investigated.

## Author Contributions

RS designed and performed research, analyzed and interpreted data, and wrote the manuscript. GF designed and performed research, and interpreted the data. MF and BQ performed research and generate the shRNA FHT transfectants. CG, CC, MD, GCon, VV, and AW performed research. GCu, NP, EG, SF, and FSC contributed to the data analysis and interpretation. KK contributed to the data analysis, interpretation, and manuscript writing. FC designed research, interpreted the data, supervised the work, and wrote the manuscript. EC conceived the study, designed research, interpreted the data supervised the work, and wrote the manuscript. All the authors read and approved the final manuscript.

### Conflict of Interest Statement

The authors declare that the research was conducted in the absence of any commercial or financial relationships that could be construed as a potential conflict of interest.

## Supplementary Material

Supplementary Figure 1 Reduced MHC class I surface expression molecules in established tumor cells and human macrophages after DFO treatment and in FTH-silenced cells. **(A)** Mel-30 and Mel-35 primary melanoma cells were treated overnight with DFO and non-classical (HLA-E) MHC class I surface expression was measured by flow cytometry. The dashed curve in the two histograms represents the isotype control; the white curve represents the untreated control (Nt) cells and the filled gray curve represents cells treated with DFO. Columns show statistical analysis of three independent experiments. *P*-values were calculated using paired Student *t-*test (^**^*p* < 0.01). **(B)** Phenotype of human macrophages treated or not with DFO. The dashed curve in the histograms represents the isotype control; the white curve represents untreated control cells and the filled gray curve represents cells treated with DFO. Columns show statistical analysis of nine independent experiments. *P*-values were calculated using paired Student *t-*test (^*^*p* < 0.05). **(C)** MM07m shCTRL (white histogram) and MM07m shFTH clone 1 (filled gray curve) and clone 2 (filled gray curve) were stained with W6/32 and the expression of MHC class I molecule was quantified by FACS. One representative experiment is shown. Quantification of the surface expression levels of MHC class I molecules or HLA-E molecules in MM07m **(C)** and MCF7 cells. **(D)** Dashed curves represent isotype control; white curves represent shCTRL cells and gray curves represent shFTH cells. Statistical analysis was obtained from six consecutive experiments. *P*-values were calculated using paired Student *t-*test (^*^*p* < 0.05; ^***^*p* < 0.001).

Supplementary Figure 2 Iron levels affect CD86 and MHC class II on human macrophages. Phenotype of human macrophages treated or not with DFO. The dashed curve in the histograms represents the isotype control; the white curve represents untreated control cells and the filled gray curve represents cells treated with DFO. Columns show statistical analysis of nine independent experiments. *P*-values were calculated using paired Student *t-*test (^*^*p* < 0.05; ^***^*p* < 0.001).

Supplementary Figure 3 Iron levels affect HLA-E up regulation by interferon-γ stimulation. **(A–C)** Mel-30 and Mel-35 primary melanoma cells were grown in presence IFN-γ, or a combination of DFO and IFN-γ. Cells were stained with non-classical MHC-class I molecule (HLA-E) or CD155 and analyzed by flow cytometry. The dashed curve in the two histograms represents the isotype control; the white curve represents the untreated control cells; the black curve represents cells stimulated with IFN-γ and the dark gray curve represents cells treated with DFO + IFN-γ. Columns show statistical analysis of three independent experiments. Statistical analysis was performed by ANOVA followed by Holm-Sidak's multiple comparisons test. ^*^*P* < 0.05; ^**^*P* < 0.01; ^***^*P* < 0.001.

Supplementary Figure 4 Iron levels regulate Macrophages NK cell recognition. **(A)** Human macrophages were tested for their susceptibility to NK cell killing after DFO treatment (gray squares) and without any treatment (white squares). One representative experiment is shown. Columns represent statistical analysis from three consecutive experiments at 25:1 and 12:1 effector:target ratio performed using paired Student *t-*test (^*^*p* < 0.05; ^**^*p* < 0.01). **(B)** Freshly isolated NK cells not treated (white squares) and treated with DFO (gray squares) were used in lymphocytotoxicity assays using K562 as target cells. The experiment was performed in triplicate. *P*-values were calculated using 2-tailed paired Student *t-*test. **(C)** FACS analysis of the surface expression levels of DNAM1 ligands (CD155 and CD112) in MM07m shCTRL (white histogram) and MM07m shFTH clone 1 (filled gray histogram). One representative experiment is shown. Dashed curves represent isotype control. **(D)** Facs analysis of the surface expression levels of NKG2D ligands in MM07m shCTRL (white bars) and MM07m shFTH clone 1 (gray bars). Statistical analysis was obtained from six consecutive experiments.
