# Supplementary figures and images for: Iron and Ferritin Modulate MHC Class I Expression and NK Cell Recognition
Source: Front Immunol. 2019 Feb 26;10:224. doi: 10.3389/fimmu.2019.00224 (PMC6404638; doi:10.3389/fimmu.2019.00224)

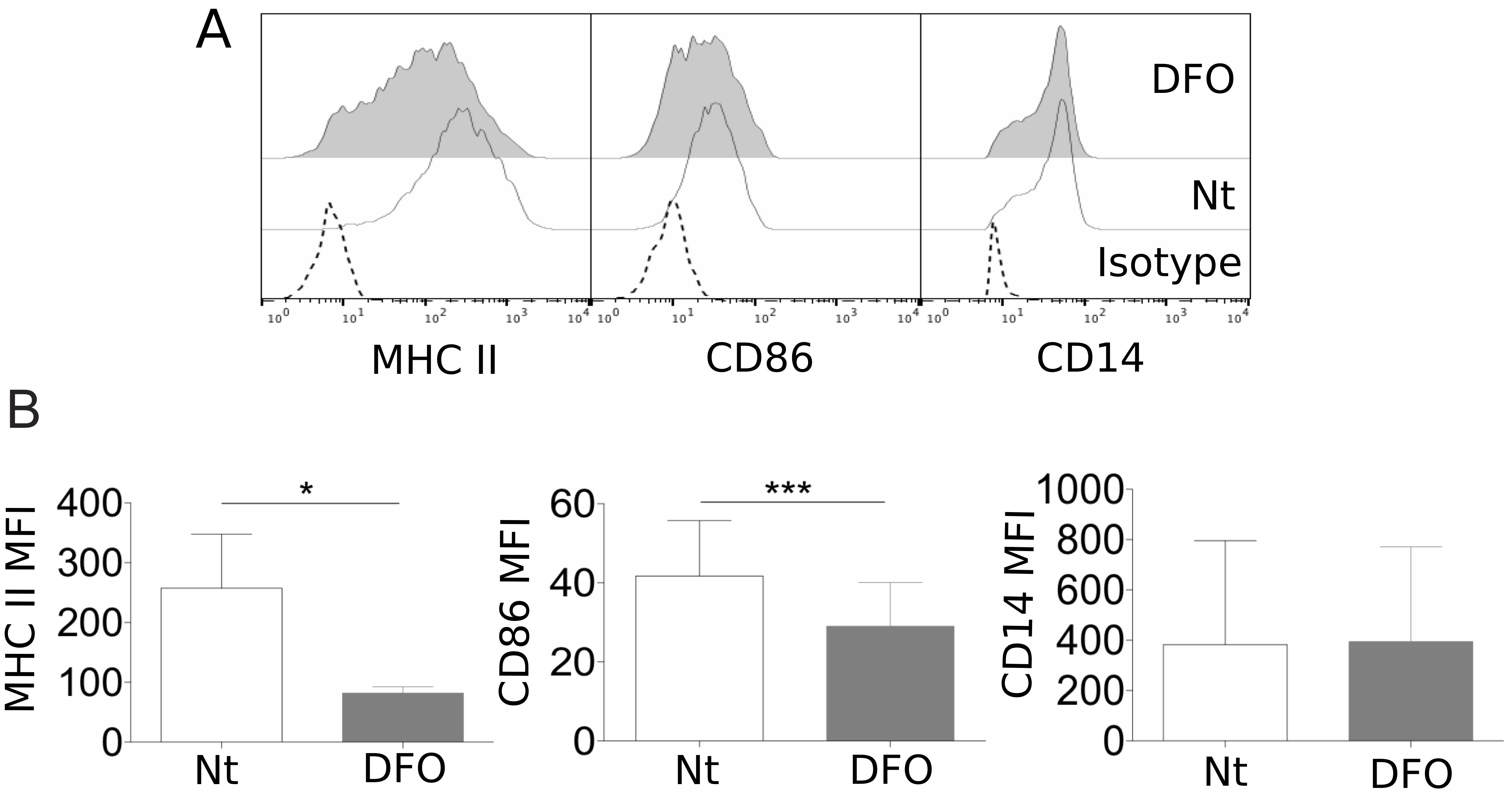

Supplement: Supplementary Figure 2 — Iron levels affect CD86 and MHC class II on human macrophages. Phenotype of human macrophages treated or not with DFO. The dashed curve in the histograms represents the isotype control; the white curve represents untreated control cells and the filled gray curve represents cells treated with DFO. Columns show statistical analysis of nine independent experiments. P-values were calculated using paired Student t-test (*p < 0.05; ***p < 0.001). [file Image_2.TIFF]

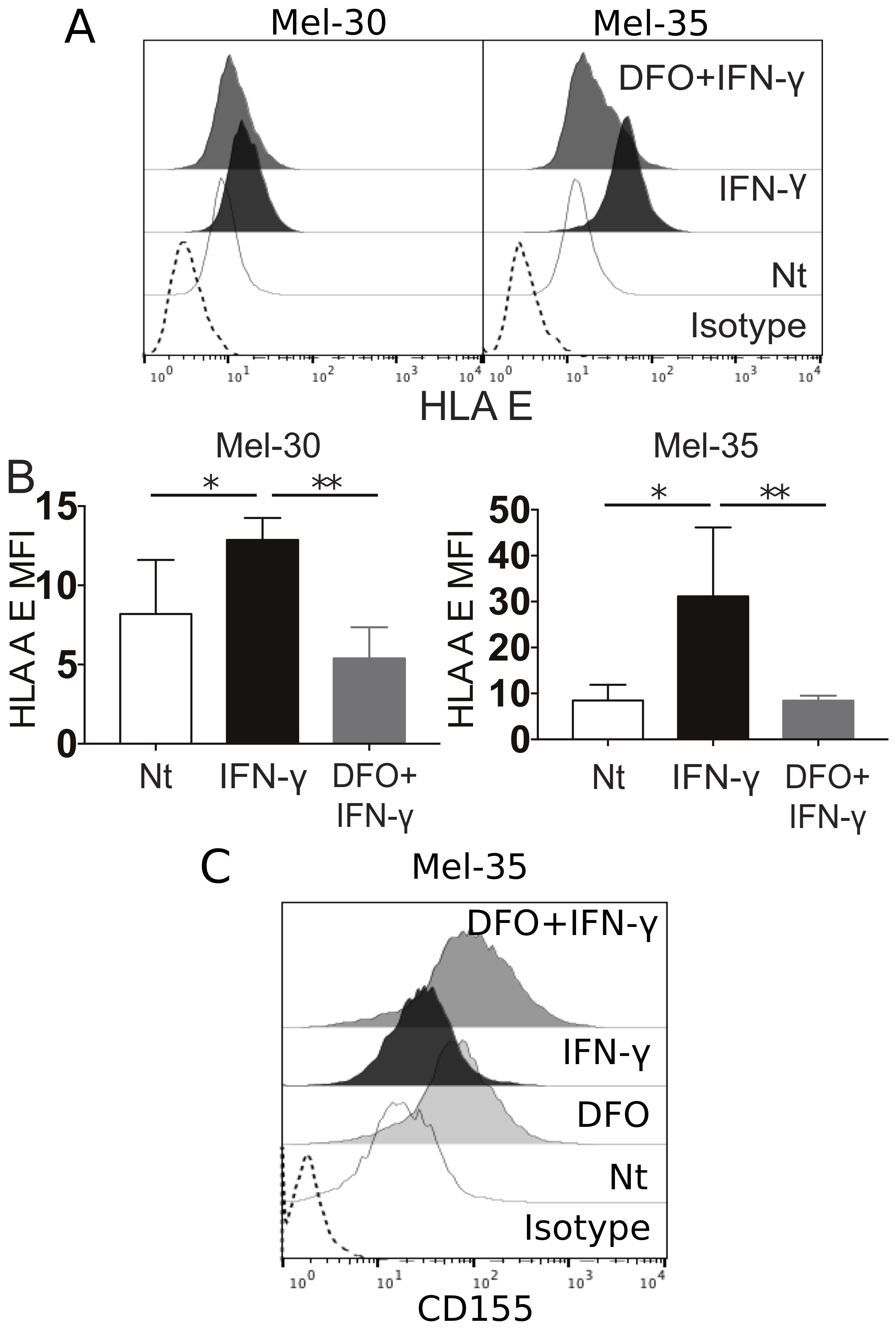

Supplement: Supplementary Figure 3 — Iron levels affect HLA-E up regulation by interferon-γ stimulation. (A–C) Mel-30 and Mel-35 primary melanoma cells were grown in presence IFN-γ, or a combination of DFO and IFN-γ. Cells were stained with non-classical MHC-class I molecule (HLA-E) or CD155 and analyzed by flow cytometry. The dashed curve in the two histograms represents the isotype control; the white curve represents the untreated control cells; the black curve represents cells stimulated with IFN-γ and the dark gray curve represents cells treated with DFO + IFN-γ. Columns show statistical analysis of three independent experiments. Statistical analysis was performed by ANOVA followed by Holm-Sidak's multiple comparisons test. *P < 0.05; **P < 0.01; ***P < 0.001. [file Image_3.tiff]

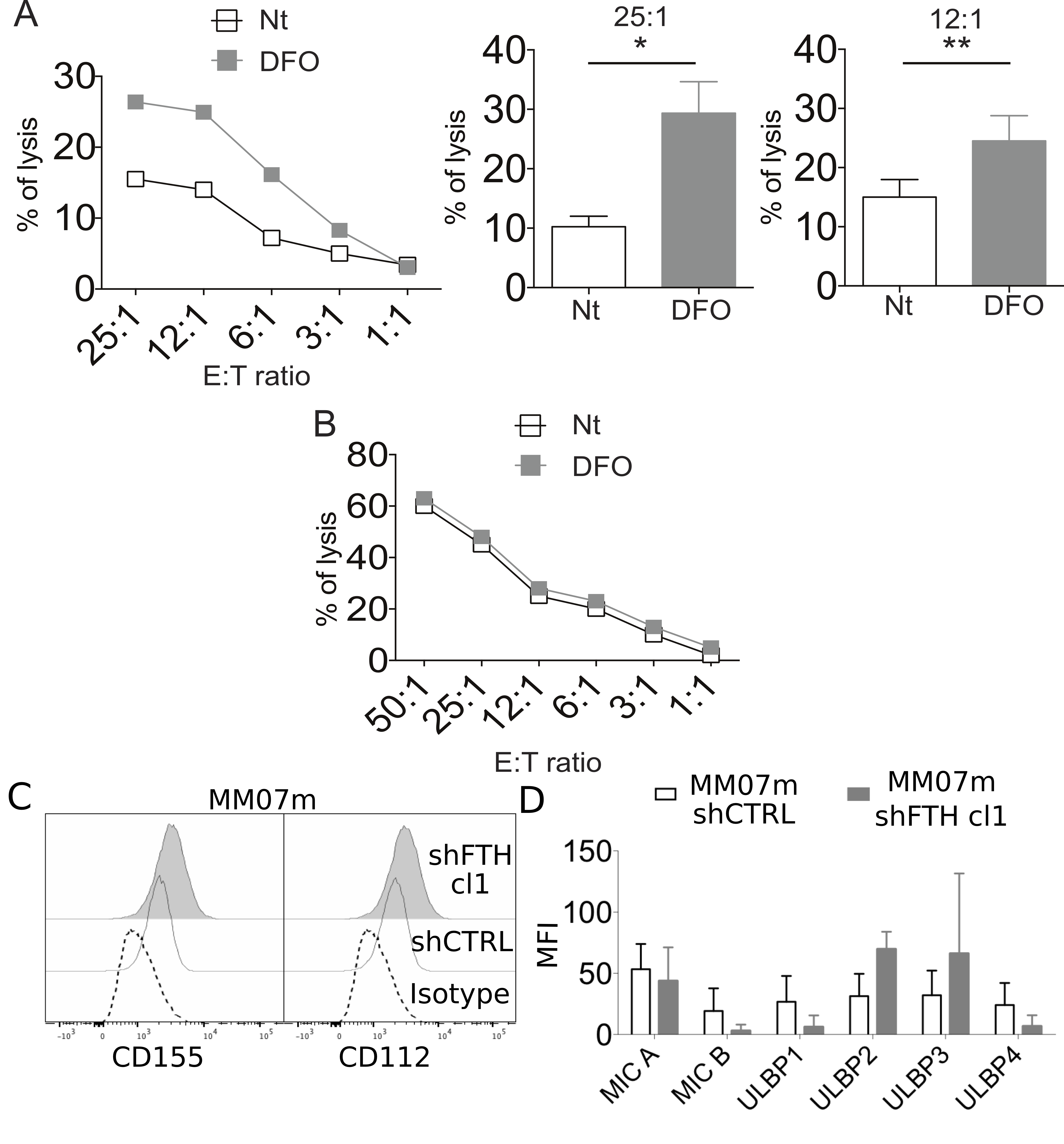

Supplement: Supplementary Figure 4 — Iron levels regulate Macrophages NK cell recognition. (A) Human macrophages were tested for their susceptibility to NK cell killing after DFO treatment (gray squares) and without any treatment (white squares). One representative experiment is shown. Columns represent statistical analysis from three consecutive experiments at 25:1 and 12:1 effector:target ratio performed using paired Student t-test (*p < 0.05; **p < 0.01). (B) Freshly isolated NK cells not treated (white squares) and treated with DFO (gray squares) were used in lymphocytotoxicity assays using K562 as target cells. The experiment was performed in triplicate. P-values were calculated using 2-tailed paired Student t-test. (C) FACS analysis of the surface expression levels of DNAM1 ligands (CD155 and CD112) in MM07m shCTRL (white histogram) and MM07m shFTH clone 1 (filled gray histogram). One representative experiment is shown. Dashed curves represent isotype control. (D) Facs analysis of the surface expression levels of NKG2D ligands in MM07m shCTRL (white bars) and MM07m shFTH clone 1 (gray bars). Statistical analysis was obtained from six consecutive experiments. [file Image_4.TIFF]
